# Genomic and Post-Translational Modification Analysis of Leucine-Rich-Repeat Receptor-Like Kinases in *Brassica rapa*

**DOI:** 10.1371/journal.pone.0142255

**Published:** 2015-11-20

**Authors:** Jana Jeevan Rameneni, Yeon Lee, Vignesh Dhandapani, Xiaona Yu, Su Ryun Choi, Man-Ho Oh, Yong Pyo Lim

**Affiliations:** 1 Molecular Genetics and Genomics Laboratory, Department of Horticulture, College of Agriculture and Life Science, Chungnam National University, Daejeon 305–764, Korea; 2 Department of Biological Sciences, College of Biological Sciences and Biotechnology, Chungnam National University, Daejeon, 305–764, Korea; NSW Department of Primary Industries, AUSTRALIA

## Abstract

Among several receptor-like kinases (RLKs), leucine-rich-repeat receptor-like kinases (LRR-RLKs) are a major group of genes that play crucial roles in growth, development and stress responses in plant systems. Given that they have several functional roles, it is important to investigate their roles in *Brassica rapa*. In the present study, 303 LRR-RLKs were identified in the genome of *B*. *rapa* and comparative phylogenetic analysis of 1213 combined LRR-RLKs of *B*. *rapa*, *Arabidopsis thaliana*, *Oryza sativa* and *Populus trichocarpa* helped us to categorize the gene family into 15 subfamilies based on their sequence and structural similarities. The chromosome localizations of 293 genes allowed the prediction of duplicates, and motif conservation and intron/exon patterns showed differences among the *B*. *rapa* LRR-RLK (*BrLRR-RLK*) genes. Additionally, computational function annotation and expression analysis was used to predict their possible functional roles in the plant system. Biochemical results for 11 selected genes showed variations in phosphorylation activity. Interestingly, *BrBAK1* showed strong auto-phosphorylation and trans-phosphorylation on its tyrosine and threonine residues compared with *AtBAK1* in previous studies. The *AtBAK1* receptor kinase is involved in plant growth and development, plant innate immunity, and programmed cell death, and our results suggest that *BrBAK1* might also be involved in the same functions. Another interesting result was that *BrBAK1*, *BrBRI1*, *BrPEPR1* and *BrPEPR2* showed activity with both anti-phosphotyrosine and anti-phosphothreonine antibodies, indicating that they might have dual-specificity kinase activity. This study provides comprehensive results for the *BrLRR-RLK*s, revealing expansion of the gene family through gene duplications, structural similarities and variations among the genes, and potential functional roles according to gene ontology, transcriptome profiling and biochemical analysis.

## Introduction

The Brassicaceae family is one of the major groups in the plant kingdom, composed of 340–360 genera and over 3,700 species distributed worldwide [1]. Among the six most cultivated species, the mesopolyploid *Brassica rapa* (AA genome, 2n = 2x = 20) is an economically, agronomically and scientifically important crop. Plants, animals and other multi-cellular organisms carry out inter- and intracellular communications with cell-surface receptor-like kinases (RLKs) through chemical signals. The first RLK was identified in maize [2] and subsequently RLKs were found in other plant species, including Arabidopsis and rice, which contain 610 and 1132 genes, respectively [3]. Furthermore, Arabidopsis RLKs were subdivided into 44 subfamilies according to phylogenetic analysis, and roughly 21 different extracellular domains were predicted [4].

Leucine-rich-repeat receptor-like kinases (LRR- RLK) form one of the major super-families in plant species and consist of an N-terminal LRR as an extracellular domain (ECD) and a C-terminal kinase domain. The LRR domain is 20–30 amino acids in length, is the major class of ECD [5] found among all of the RLKs in plant species, and is predicted to be involved in protein–protein interactions [6]. Additionally, LRR motifs comprise at least eight different classes with varying numbers and arrangements of LRRs in the sequence [4,5]. The protein kinase domain forms a lobe structure that consists of approximately 250–300 amino acids and has two sub-divisions, protein-serine/threonine kinases and protein tyrosine kinases. It is also divided into twelve sub-domains that are highly conserved and involved in catalytic activity, specifically in functions related to cell development [7]. Previously, these genes were predicted to be involved in different functions and placed in three different groups: (1) plant organ growth and development, (2) functions related to biotic and abiotic stresses and (3) multiple functions. For example, In *A*. *thaliana*, *SOMATIC EMBRYOGENESIS RECEPTOR-LIKE KINASE* (*SERK*) is involved in anther development and disease resistance [8], *ERECTA* (*ER*) functions in both ovule development and bacterial wilt [9,10], *STRUBBELIG-RECEPTOR FAMILY* (*SRF*) that functions in development of organs like leaves, stem and flower [11], *FLAGELLIN-SENSITIVE* 2 (*FLS*2) and *PEP1 RECEPTOR* (*PEPR*) is involved in both pathogen recognition and defense response and similarly, *EF-TU RECEPTOR* (*EFR*) is involved in enhancing immunity against few bacterial disease in rice, [12, 13, 14]. In addition, *PHYTOSULFOKIN RECEPTOR 1* (*PSKR1*) is involved in cell proliferation and also in defense response against bacterial pathogens in Arabidopsis [15], *BAK1-INTERACTING RECEPTOR-LIKE KINASE 1* (*BIR1*) is involved in both cell death and plant growth in temperature dependent manner [16], and *BRASSINOSTEROID INSENSITIVE* (*BRI1*) along with BRI1 ASSOCIATED RECEPTOR KINASE1 (BAK1) is involved in Brassinosteroid signaling [17]. Generally, Kinases that are involved in phosphorylation are conserved with positively charged arginine (R) and negatively charged aspartate (D) amino acid residues at the activation site, based on the conservation of the arginine (R) residue these kinases are divided into RD and non-RD type [18,19]. Likely, RD kinases are capable of auto-phosphorylation of the stimulation loop that is necessary for kinase activity. Conversely, non-RD kinases are mainly involved in plant immunity against microbes [18,19].

In this work, the whole genome sequence (WGS) of *B*. *rapa* [20] was used to identify *B*. *rapa* LRR-RLK (*BrLRR-RLK*) genes genome-wide. As these genes are important for functions related to plant morphological characters and plant stress resistance [5], characterizing this gene family will be useful for the genetic improvement of *B*. *rapa*. Because of the breeding and scientific importance of Chiifu (Chinese cabbage) among all the Chinese cabbage varieties, it was used for the identification of 303 *BrLRR-RLK* genes in the *B*. *rapa* genome. Furthermore, using these genes along with LRR-RLK genes from rice, Arabidopsis and *Populus*, a comparative phylogeny was constructed and we determined the motif patterns and intron/exon structures for all of the genes. Additionally, the computational functional annotations, the expression patterns among six tissues [21] and the biochemical activity of selected genes were also investigated.

## Materials and Methods

### Identification, mapping, and duplication analysis of LRR-RLK genes in *B*. *rapa*

Previously published *Arabidopsis thaliana* LRR-RLK protein sequences were retrieved and these genes were submitted to a Pfam batch search (http://pfam.sanger.ac.uk/search#tabview=tab1) to find the Pfam IDs. A protein alignment was retrieved for the Pfam IDs (PF00560, PF08263, PF12799, PF13516, PF13855, PF00069, PF07714) in the Stockholm format from the Pfam database (http://pfam.xfam.org/family/pf00069#tabview=tab3) and using this as a reference, we searched for LRR-RLKs among the protein sequences of *B*. *rapa* downloaded from the BRAD database (http://brassicadb.org/brad/). Redundant sequences were removed using our own Perl script and further confirmed by manual and Pfam batch searches. The BLAST-like Alignment Tool (BLAT) and TBlastN software were employed to map the positions of the LRR-RLK genes on the *B*. *rapa* chromosomes. Multiple sequence alignment and comparative analysis were performed to identify the duplicated *BrLRR-RLK* genes in the whole genome of *B*. *rapa*. The candidate genes were named *BrLRR-RLK1* to *BrLRR-RLK303* based on their positions counted from top to bottom on chromosomes A01 to A10. An *A*. *thaliana* ortholog search was carried out with the mega BLAST tool, which compared the full coding sequences of the *BrLRR-RLK* genes and all available *A*. *thaliana* genes. Highly similar (e-value > e-45) homologous pairs were filtered out with in-home developed Perl scripts and further the resulted orthologs were cross checked with BRAD database and considered as orthologous genes.

### Intron/exon analysis

The evolution and functions of a particular gene can be predicted through intron and exon patterns. Thus, to determine the intron and exon distribution and splicing phases of the 303 *BrLRR-RLK*s, we compared the *BrLRR-RLK* coding sequences (CDSs) and corresponding genomic sequences using the online Gene Structure Display Server (GSDS) (http://gsds.cbi.pku.edu.cn).

### Conserved motifs and gene ontology annotation

The Multiple Expectation Minimization for Motif Elicitation (MEME) tool (http://meme.nbcr.net/meme/cgi-bin/meme.cgi) [22] was used with default parameters to identify the conserved motifs within the 303 *BrLRR-RLK* protein sequences. A maximum of 25 motifs were searched and the types of motifs were determined using the Simpler Modular Architecture Research Tool (SMART) (http://smart.embl-heidelberg.de/) and a Pfam batch search (http://pfam.sanger.ac.uk/search#tabview=tab1) [23]. Additionally, basic information for the *BrLRR-RLK* protein sequences like PI, length (aa) and molecular weight was collected using the sequence manipulation suite (SMS) V. 2 (http://www.bioinformatics.org/sms2/index.html). To identify *BrLRR-RLK* gene divergence, signal peptides and transmembrane domains were predicted using the online tool Phobius (http://phobius.sbc.su.se/) [24].

The molecular function, biological process and cellular component annotations were mapped from plant proteins that had high-scoring segment pairs (HSPs) with the *BrLRR-RLK* proteins in a BlastP alignment using the Blast2Go software and the data was retrieved in the text file format.

### Phylogenetic tree construction

Using the ClustalX-2.0 software package with default parameters [25], multiple sequence alignments were performed with the LRR-RLK protein sequences of *A*. *thaliana*, *B*. *rapa*, *Oryza sativa*, and *Populus trichocarpa*. The neighbor-joining (NJ) method with 1000 bootstrap iterations was used for phylogenetic tree construction with the aligned LRR-RLK protein sequences. A circular phylogenetic tree was generated using the Molecular Evolutionary Genetics Analysis (MEGA) V5.1 software package (http://www.megasoftware.net/mega.php) [26].

### Heat map expression pattern of LRR- RLK genes in *B*. *rapa* using RNA-seq data

For *BrLRR-RLK* gene expression analysis, RNA-seq data under accession no. GSE43245 previously produced and analyzed by Tong et al. [21] were downloaded from the NCBI Gene Expression Omnibus database (http://www.ncbi.nlm.nih.gov/geo/). These data included six tissues of *B*. *rapa* accession Chiifu-401-42: callus, root, stem, leaf, flower, and silique. Transcript abundance was recorded as fragments per kilobase of exon model per million mapped (FPKM), and a heat map of the *BrLRR-RLK* genes was generated using the CIMminer online software (http://discover.nci.nih.gov/cimminer/oneMatrix.do).

### Cytoplasmic domain construction for recombinant proteins

Total RNA was extracted from leaf tissues of *B*. *rapa* Chiifu (Chinese cabbage) using TRI reagent according to the manufacturer’s protocol (Applied Biosystems, Carlsbad, CA, USA) [27] and 2 μg of DNase-treated RNA was reverse transcribed into single-strand cDNA with Superscript III reverse transcriptase (Applied Biosystems). Eleven predicted *BrLRR-RLK* gene sequences from the Brassica database were compared by BlastN with the Arabidopsis genome information (AGI) CDS dataset to confirm their sequence identity. Then, the 11 selected genes were synthesized using gene-specific primers (S1 Table) and Accuprime pfu DNA polymerase (Applied Biosystems) with PCR conditions of 95°C for 2 min, 35 cycles of 95°C for 20 sec, 60°C for 40 sec, and 72°C for 2 min, with a final 72°C for 5 min. The amplified fragments were purified and cloned into the pENTRY-TOPO vector (Applied Biosystems) and confirmed as *BrLRR-RLK* genes through sequence analysis. Recombinant Flag-*BrLRR-RLK*s were amplified using pFLAG-MAC (Sigma-Aldrich, St Louis, MO, USA) and the PCR products were digested with specific enzymes and purified by agarose gel electrophoresis. The resulting fragments were ligated into pFLAG-MAC, which was digested with enzymes, followed by heat shock transformation into *E*. *coli* (DH5α) and plasmid extraction. The mutations were confirmed by sequence analysis.

### Recombinant protein and immunoblot analysis

Flag-*BrLRR-RLK*-CDs were expressed in BL21 (DE3) pLysS *E*. *coli* cells (Novagen, Merck, Darmstadt, Germany) and cultures were induced with 0.3 mM IPTG at 23°C for 16 h. Flag-*BrLRR-RLK*-CD cell lysate preparation and *in vitro* phosphorylation assay conditions were as previously described [28]. Recombinant Flag-*BrLRR-RLK* cytoplasmic domain proteins were subjected to SDS-PAGE, transferred to polyvinyl difluoride membranes, and immunoblot analysis was carried out using anti-Flag antibodies (1:5,000 dilution), anti-phosphothreonine antibodies (1:500 dilution), and anti-phosphotyrosine antibodies (1:500 dilution). Protein quantification was done using Coomassie Brilliant Blue staining and immunoblots were scanned using an Odyssey Infrared Imaging System (LI-COR Bioscience, Lincoln, NE, USA) for visualization.

## Results and Discussion

### LRR-RLK gene identification and chromosomal locations in the *B*. *rapa* genome

**Fig 1:**
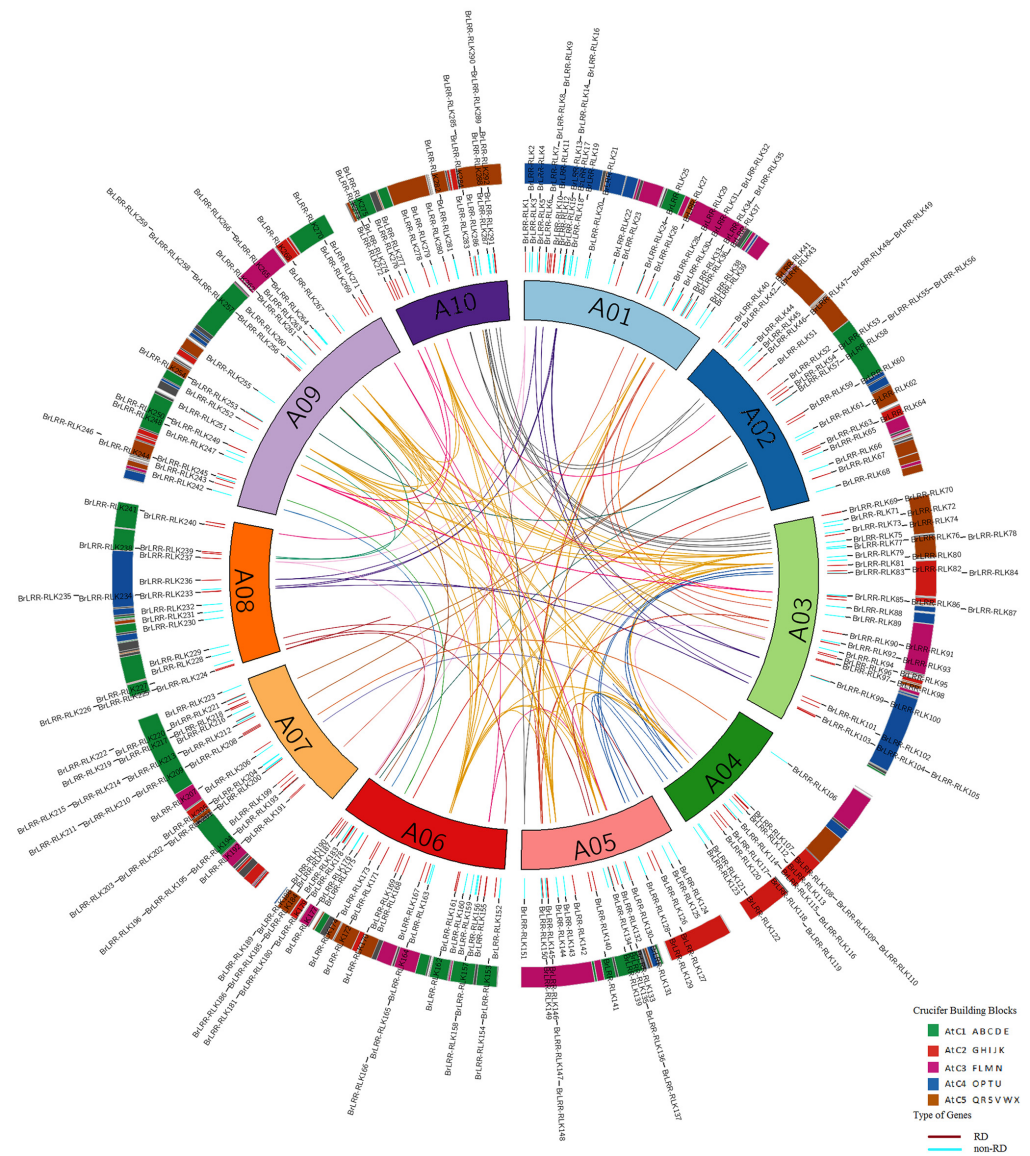
Chromosomal distribution of 293 *BrLRR-RLK* genes. Outer circle with colored blocks illustrates different crucifer building blocks on chromosomes. The RD (red color) and non-RD (aqua color) type genes are distributed on chromosomes A01 to A10 in inner circle and paralogous copies are marked with different colored lines in center.

A total of 303 non-redundant *BrLRR-RLK* genes were identified in the *B*. *rapa* genome and were confirmed through manual and Pfam searches for the presence of both ECDs and kinase domains (KDs) in the sequences. The homologous *LRR-RLK* genes identified in *B*. *rapa* in our study are reported in *A*. *thaliana*, *O*. *sativa* and *P*. *trichocarpa* to have important functional roles related to plant growth and development [29–32]. Most of the *BrLRR-RLK* genes contained leucine rich repeats (LRRs) in their ECDs, which play a major role in protein–protein interactions [6], whereas transmembrane (TM) domains and signal peptides (SP) are signatures of membrane RLK proteins [5]. In total, 293 genes, *BrLRR-RLK1* to *BrLRR-RLK293*, were physically located on the 10 chromosomes and whereas the remaining 10 genes, *BrLRR-RLK294* to *BrLRR-RLK303* were mapped on scaffolds using the BLAT and TblastN software. The *BrLRR-RLK* genes were randomly distributed among all 10 chromosomes, with A01 and A06 having the most (39 genes) and A10 having the least (10 genes) (Fig 1 and Table 1).

**Table 1:** The complete information and characteristics of *B*. *rapa LRR-RLK* genes.

**Chromosome**	**Gene number**	**Groups**	**Type**	**Intron number**	**Length (aa)**	**PI**	**MW(KDa)**	**SP Number**	**Genes with TM**	**Homologous Arabidopsis genes**
A01	39	I-VII, VIII-2, IX-XIII	RD, Non-RD	0–21	516–1554	5.05–9.17	58.12–170.68	1–3	27	BIN1, SERK3, MRH1, GSO1, BAM3, SRF8, IMK2, MEE39, NIK3, IKU2, SRF7, SRF3
A02	29	I-III, V-XIII	RD, Non-RD	0–23	515–1465	5.09–8.59	57.29–160.45	1	21	PSY1, FLS2, PSKR1, BIR1
A03	37	I-III, V-XIV	RD, Non-RD	0–22	479–1035	5.06–9.81	53.51–113.58	1–2	27	SRF2, NIK1, EFR, SOBIR1, FEI2, SRF3, TMK1, SERK4, BAM2, BAM3
A04	18	I-IV, VII, X-XIII	RD, Non-RD	0–18	487–1564	5.81–10.41	54.52–175.14	0–2	15	MRLRR- RLK, SERK4, SOBIR1, FEI2
A05	28	I, III-V,VIII-2, X-XIII	RD, Non-RD	0–22	490–1095	5.01–9.12	54.09–119.42	0–1	22	FEI2, SRF7, BRL3, SRF4
A06	39	I-III,V-VII, VIII-2, IX-XIV	RD, Non-RD	0–23	537–1785	4.87–10.21	60.17–195.16	1–2	37	SRF6, SRF9, PEPR2, BAM1, ERL1, NIK2, VH1, MEE62, GSO2, ATBRI1
A07	33	I-III,V, VII,VIII-2,IX-XI	RD, Non-RD	0–22	554–1138	4.85–9.15	60.62–123.25	1–2	23	HSL1, MRLRR- RLK, PEPR1, SERK1, TMK1, PSY1, FLO5, SRF5
A08	18	I-III, V, VIII-2, X,XI	RD, Non-RD	0–23	566–1174	4.79–8.96	62.83–128.33	1–2	14	BRL1, SRF6, BAM3, SERK3, DWF2A, HSL1, CLV1
A09	30	I-III,VI-XIII	RD, Non-RD	0–24	300–1994	4.83–8.88	33.05–222.23	0–2	24	HSL2, HSL1, CRK41, FLS2, QRP1
**Chromosome**	**Gene number**	**Groups**	**Type**	**Intron number**	**Length (aa)**	**PI**	**MW(KDa)**	**SP Number**	**Genes with TM**	**Homologous Arabidopsis genes**
A10	22	I-III, V, VI, VIII-1, X-XIV	RD, Non-RD	0–22	597–1233	5.32–9.62	66.28–137.67	0–2	18	EFR, NIK1, SRF2, EXS, ERL2
Scaffolds	10	II, III, V, VIII-2, XI,XII	RD, Non-RD	1–23	517–1243	5.17–8.69	57.16–135.62	0–2	6	SERK4, SRF4, GSO2

PI, isoelectric point; aa, amino acids; MW, molecular weight; SP, signal peptide; TM, transmembrane.

Among non-RD types, the highest number (23 genes) was found on chromosome A01 and the remaining 109 genes were distributed randomly among the other chromosomes including scaffolds. In the case of RD type genes, 25 genes were mapped on A06, followed by 21 on A03, and the least number (4 genes) were found to be on scaffolds (S2 Table). Orthologous genes are homologous genes found in two different species but which originated from a common ancestor, and if a gene is duplicated and occupies two different positions in the same genome, then the two copies are considered paralogous [33]. In our analysis, around 143 genes were duplicated and formed 143 paralogous copies (Fig 1 and S3 Table). We also identified 11 *BrLRR-RLK* genes that were not orthologous to *A*. *thaliana* LRR-RLK genes (S2 Table). Surprisingly, 31 *BrLRR-RLK* genes were missing from crucifier genome building blocks that were confirmed by computational and manual curation, whereas the remaining 272 genes were categorized under 23 genomic blocks (GB) with a maximum of 30 in GB “U” and a minimum of 1 in “G”; moreover, 35 and 12 *BrLRR-RLK* genes have undergone tandem and segmental duplications (Fig 1, S2 Table and S3 Table). These results indicate that the duplication and uneven distribution of the genes on the chromosomes and GBs occurred because of duplication events during the process of evolution [34–36].

### Comparative phylogenetic analysis of LRR-RLK genes in *A*. *thaliana*, *B*. *rapa*, *O*. *sativa*, and *P*. *trichocarpa*

**Fig 2:**
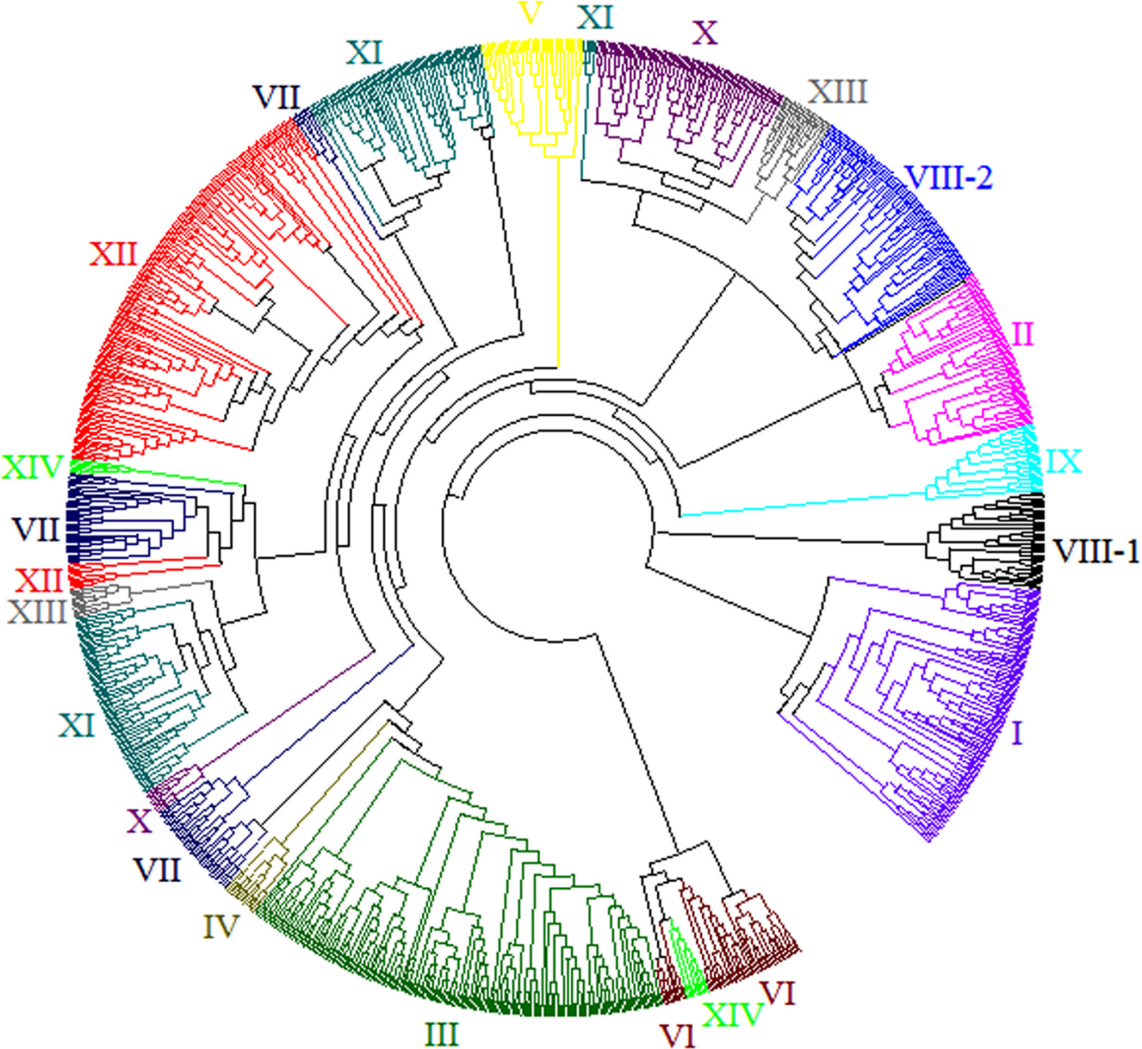
Comparative phylogenetic analysis of LRR-RLKs of *B*. *rapa*, *A*. *thaliana*, *O*. *sativa and P*. *trichocarpa*. The circular tree was generated using MEGA5.0 with 1213 genes through the neighbor-joining method. The genes were divided into 14 clusters (I to XIV) indicated in different colors.

To identify the evolutionary relationships between the *BrLRR-RLK*s and other species, multiple alignments of 222, 303, 309 and 379 LRR-RLK proteins of *A*. *thaliana*, *B*. *rapa*, *O*. *sativa* and *P*. *trichocarpa*, respectively, were used for comparative phylogenetic analysis. The 1213 LRR-RLK proteins were divided into 15 subfamilies (I to XIV) according to the node support between the LRR-RLK genes [4,29,37] and to differentiate the subfamilies, each was indicated with a random color (Fig 2 and S1 Fig). Subfamily XII contained the most genes (186), followed by subfamilies III (181), XI (162), I (117), X (92), VII (88), VIII-2 (86), II (68), VI (52), V (43), VIII-1 (41), XIII (35) and IX (29), with the least number of genes in subfamilies XIV and IV (16 each) (S1 Fig). The origin of the RLK genes was predicted to be before the speciation of plants, animals, fungi and protists but expansion of this super-family might have occurred throughout land plant evolution [4,37]. The LRR-RLK genes of *B*. *rapa* and *A*. *thaliana* were evenly distributed among all the subfamilies but the rice and *Populus* LRR-RLK genes formed monophyletic clusters consisting of species-specific genes [30,38]. Similarly, monocot and dicot LRR-RLK genes were observed in all the subfamilies, indicating that expansion of the family might have occurred before their divergence [39]. Additionally, in our comparative phylogenetic analysis we observed that all the subfamilies comprised 30–80% monocot genes, but most contained 50–60% monocot genes. Surprisingly, subfamilies XII and VII contained the least number of dicot genes (20% and 25%, respectively). Comparison between monocot and dicot LRR-RLK gene numbers among the subfamilies revealed that gene duplication might have occurred largely within subfamilies XI in monocots and dicots, III and I in dicots and XII in monocots (S1 Fig). Since sister pairs denote the closest relatives of a group in a phylogenetic tree, we analyzed and predicted 342 pairs, this covered 56.3% of the total number of genes used in this study. These sister pairs included 288 pairs in the same species and 154 cross-species pairs, among which the highest number of cross-species pairs was 149 (*AtLRR-RLK- BrLRR-RLK*) (S2 Fig) and the least number was 1 (*PtLRR-RLK- BrLRR-RLK*). In the case of same-species pairs, the most pairs (140) were present in *P*. *trichocarpa* (*PtLRR-RLK- PtLRR-RLK*) followed by 92 in rice (*OsLRR-RLK- OsLRR-RLK*), 48 in *B*. *rapa* (*BrLRR-RLK- BrLRR-RLK*) and 8 in *A*. *thaliana* (*AtLRR-RLK- AtLRR-RLK*). Same-species sister pairs were observed in all the subfamilies but the cross-species pairs, except for *AtLRR-RLK-BrLRR-RLK*, were restricted to subfamilies III and I; furthermore, the maximum bootstrap values were found among these sister pairs (S1 Fig).

Additionally, subfamilies II, VIII-1, IX, XIII and XIV consisted of RD type *BrLRR- RLK* genes and subfamilies III, IV, V and XII were composed of non-RD type genes, while subfamilies I, VI, VII, VIII-2, X and XI had both RD and non-RD type *BrLRR-RLK* genes (S2 Table). Non-RD kinases are known to function mainly as pathogen recognition receptors (PRRs) while RD kinases are involved in activation of the non-RD kinases [19,40]. Moreover, in our phylogeny we determined some of the important RD and non-RD LRR-RLK genes in different subfamilies that are involved in several functions related to plant growth and development, defense and plant immunity (S2 Table) [29,30,41].

### Intron/exon structure and duplication analysis of *BrLRR-RLK* genes

**Fig 3:**
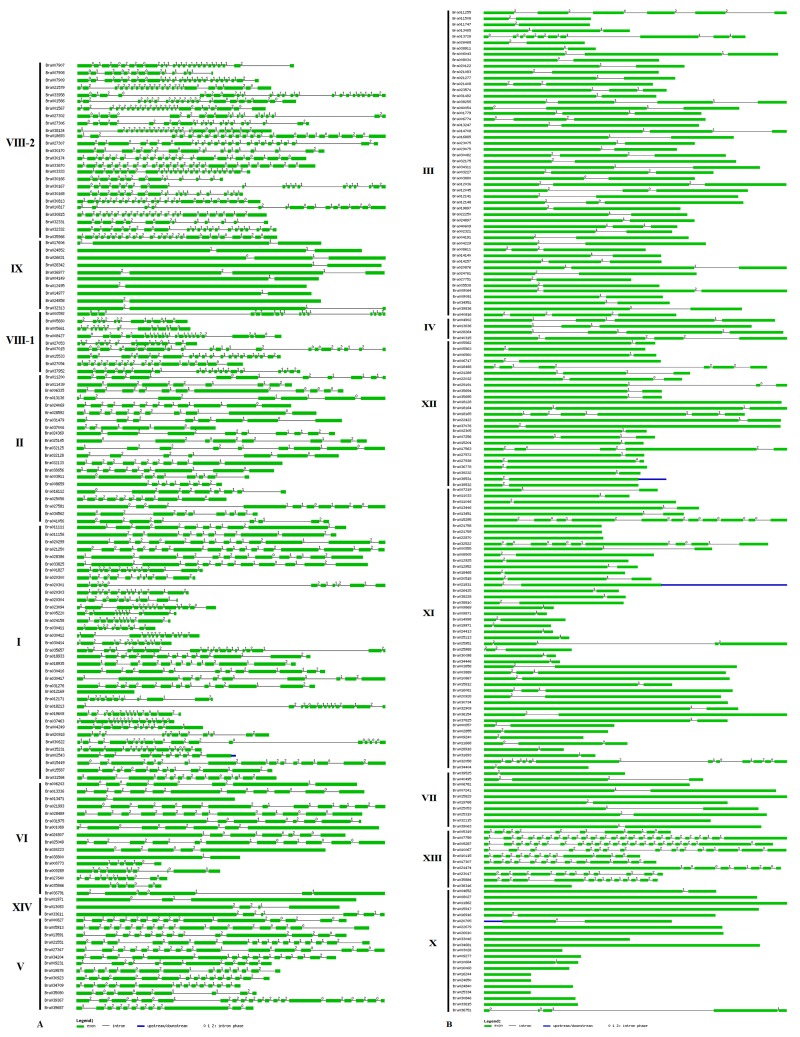
Intron/exon distribution of the 303 *BrLRR-RLK*s. (A) Subfamily (VIII-2 to V), (B) Subfamily (III to X). The genes consist of intronic regions (black line) and/or exonic regions (green line), and blue denotes regions upstream/downstream of the genes and splicing phases: 0 refers to phase 0, 1 to phase 1, and 2 to phase 2.

Intron/exon patterns can be used as a source of information for predicting the evolutionary relationships and functions of genes, so we used the genomic and coding sequences of the 303 *BrLRR-RLK* genes to identify intron/exon structure. Variations in intron/exon patterns among the subfamilies were clearly observed, irrespective of RD or non-RD type *BrLRR-RLK* genes. Intron position conservation and intron density varies widely in eukaryotic genes [42]; accordingly, in our analysis all 15 subfamilies of *BrLRR-RLK* genes showed varying intron patterns. The highest number of introns, 24, was observed in a gene (*BrLRR-RLK267/Bra007759*) of subfamily XIII, the remaining genes of which contained between 0 and 22 introns. This was followed by subfamilies VIII-2 (12–23 introns), VIII-1 (12–21), I (0–18), V (8–15), II (5–10), VI (1–10), XI (1–10), III (1–9), XIV (2–5), XII (1–5), X (0–4), IV and IX (1–3), and VII (0–2) introns. Even though subfamilies VII, IV, IX, X, XII, III and XI had 2, 3, 3, 4, 5, 9 and 10 introns at maximum, most *BrLRR-RLK* genes in these subfamilies had only one exon or 1–2 introns (Fig 3A and 3B, S2 Table).

Among the important RD and non-RD *BrLRR-RLK* genes, *SRF2*–*9*, *FEI2*, *ERL1*, *RHS16*, *MRH1* and *MEE39* had 10 or more introns, *SERK1/3/4*, *TOAD2* and *NIK1/2* had 5–10 introns, and *TMK1*, *IMK2*, *TMKL1*, *MRLK*, *PRK2*, *FLS2*, *EFR*, *GSO2*, *SOBIR1*, *BAM1*–*3*, *IKU2*, *PEPR1/2*, *FLO5*, *CLV1*, *HSL1/2*, *MEE62*, *BRI1*, *PSKR1*, *BIR1*, *BRL1/3*, *VH1* and *EXS* had fewer than five introns and several genes had only one exon (Fig 3A and 3B, S3 Table). Similarly, previous studies have been shown that the introns influence gene expression through Intron Mediated Expression (IME) and Intron-Dependent Spatial Expression (IDSE) and a study on ERECTA gene given that multiple introns in specific location enhances its expression [43–45]. In general, position of a intron indicates its conservation and site of gene expression in a plant [42,43], so in this study we performed manual calibration and found that almost all of the *BrLRR-RLK* genes had a greater number of splicing phase 2 introns, indicating a highly prone site for mutation in the intron and much less conservation. Intron phase 1 is intermediate and was the second most common phase found among the genes, but phase 0, which indicates highly conserved introns, was rare among these genes [46]. The results of our study, number of introns and splicing phases can be used further as a resource for identifying the affect of introns on *BrLRR-RLK* genes in different tissues.

### Conserved motif identification in *B*. *rapa* LRR-RLK genes

**Fig 4:**
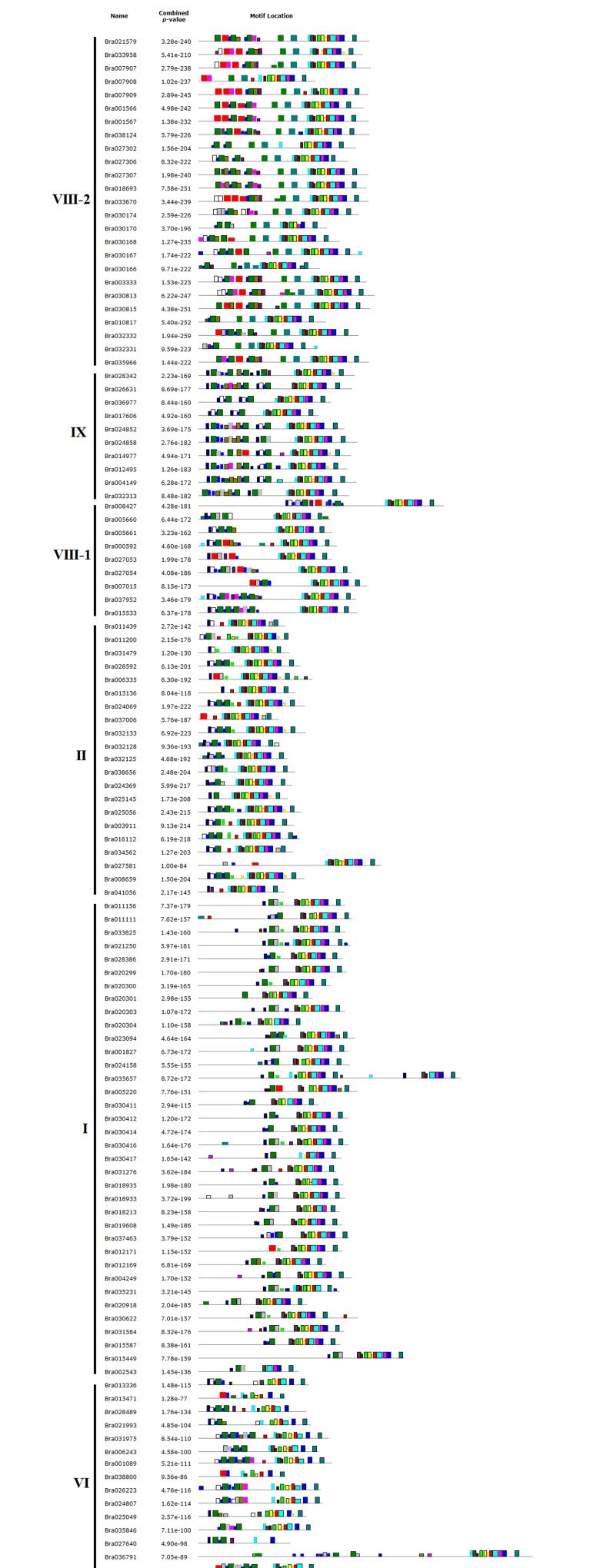
Motif distribution among the *BrLRR-RLK* genes. Twenty-five motifs in these genes were identified using the MEME search tool and grouped according to the phylogenetic tree. Each color indicates a different motif as shown at the bottom of the figure. Please refer to S5 Fig for the full size version of Fig 4.

The 303 *BrLRR-RLK* genes were divided into different groups (I–XIV) (S3 Fig) based on the phylogeny [4,5,37] and 25 motifs were identified through the MEME online tool and used to annotate the domain types. In total, 10 LRR motifs (9–13, 15, 17–19 and 25) were found in the *BrLRR-RLK* genes, with a common motif pattern among most of the genes in a subfamily and variation among the subfamilies. All 11 protein kinase motifs (1–8, 14, 16 and 20) were found in most of the genes in subfamilies II, IV, V, VII, VIII-1, VIII-2, IX, X and XIII but the remaining families did not have all 11 kinase motifs, which may have been because of the kinase domain size (Fig 4). Some additional motifs were also identified. The DUF1191 motif (22) was restricted to subfamilies VIII-2, VIII-1 and I. Similarly, the melactin motif (24) was identified in the genes of VIII-2 and one gene each in subfamilies VIII-1 and VI. Furthermore, unknown motifs (21, 23) were observed in subfamilies III, IV, XI, VII, XIII and X; motif 21 was found in II, I, VI and XII, and motif 23 was in V, XIII and XIV (Fig 4 and S4 Fig). These results support our *BrLRR-RLK* subfamily classification based on the phylogeny and also suggest that genes with different sets of protein domains in their sequences may have subfamily-specific functions in plant growth and development [29,37,39,47]. In addition, the motifs had varying lengths (11–36 aa) and numbers of sites (24–297), which are shown in Table 2. In the case of LRR motif types, the 303 *BrLRR-RLK* genes mostly contained LRR_8, followed by the leucine-rich-repeat N-terminal domain (LRRNT), LRR_6 and LRR_4, whereas the protein kinase motifs were mixed with protein kinase and protein kinase_Tyr (tyrosine kinase) [3,7] (Table 2).

**Table 2:** The amino acid patterns and characteristics of the 25 motifs identified in the 303 *BrLRR-RLK* genes.

Motif	Sequence	Length(aa)	Sites	Type
Motif1	PI[V/I]HR[D/N][I/V/L]K[S/A/P]SN[I/V]LLDED[F/L]E[A/P]K[V/L/I][S/A]DFGL[A/S][K/R]	29	281	Kinase domain
Motif2	EKSDVYSFG[V/I][V/L]LLE[L/I][L/I/V]TG[K/R][R/K][P/A]	21	297	Kinase domain
Motif3	KIAL[G/D][A/V]ARGL[A/E]YLH[E/H]	15	294	Kinase domain
Motif4	H[V/S][S/T][T/S]xV[A/R]GTIGY[I/L][A/D]PEYAx	19	199	Kinase domain-Tyr
Motif5	[D/E]E[R/K]LL[V/I]YE[Y/F]MPNGSL	15	289	Kinase domain-Tyr
Motif6	AE[V/I]Ex[L/I][GS][R/K][I/L/V]RH[R/P]NLVKL	17	293	Kinase domain-Tyr
Motif7	C[T/V]xxSPxxRP[T/S]MS[E/Q]VVxMLEE	21	292	Kinase domain
Motif8	[S/E]AN[V/I][L/I]GKGGFGTVY[K/R][G/A]VL	18	283	Kinase domain
Motif9	[S/T]GEIPS[S/E][L/I]GNLTSLxxLDLSNNx[L/F][T/S]GxIP	29	298	LRR-8
Motif10	GNLTSLxVL[D/N]LSNNN[L/F][S/T]G[S/E]IP	21	281	LRR-8
Motif11	xxL[D/N]LSYNN[L/F][S/T]GxIP	15	243	LRR-8
Motif12	GNLTSLTxLDLSNNx[F/L][S/T]Gx[I/L]P	21	223	LRR-8
Motif13	x[D/S]PxPC[S/N]WTG[VI]TC	13	229	LRRNT
Motif14	[D/N]GTxVAVK[R/K]LS	11	289	Kinase domain-Tyr
Motif15	xR[V/L]TS[L/I]DLS[G/N]NxL[S/T]GS[I/L][P/S]PE[I/L]	21	214	LRR-4
Motif16	RF[S/T][Y/L]x[E/D]LxxAT[N/D][N/G]FS	15	156	Kinase domain-Tyr
Motif17	NLx[N/S]LQxLDLSNNx[F/L]	15	257	LRR-8
Motif18	xNxL[T/S]GE[I/L]Px[S/E][L/I/F]GNLTSLxxLDLSxNxL[T/S]GEIPxE[L/I]	36	116	LRR-4
Motif19	GN[C/L]TSLxxLD[L/V]SxNRL[S/T]GE[LI]P	21	144	LRR-6
Motif20	GxxPLDWxTRL	11	257	Kinase domain-Tyr
Motif21	FxGNPGLCGxPLxxC	15	133	Unknown
Motif22	[N/M][V/I][T/S][E/D/N][N/G/H][Y/K]LEI[R/H]L[F/Y]WAGKGT[C/Q/T]C[I/L]PI[R/Q]G[N/V/A/T]YGPLIS	32	24	DUF1191
Motif23	LDLSNNxL[T/S]GPIPxE	15	139	Unknown
Motif24	AR[L/R]S[A/P][S/L/I]SL[R/T/V]YY[A/G][L/F][C/G]L[E/G]NG[G/N]Y[T/N]V[K/T/N]L[H/Q]F[A/M]EI	29	24	Melactin
Motif25	[G/S]NLxSLEYL[N/D]LSxNN[F/L]SGS[I/V/L]P	21	119	LRR-4

aa, amino acids

In general, kinases are divided into two types, arginine-aspartate (RD) and non-arginine-aspartate (non-RD), based on the conservation of the amino acid Arg (R) in their sub-domains [48]. In this study, we identified 171 RD type and 132 non-RD *BrLRR-RLK* genes based on the conservation of the ‘RD’ amino acids (S2 Table and S4 Table). Similarly, previous results suggest that the percentage of non-RD type kinases is low in Arabidopsis and rice, and, moreover, this type of kinase plays a crucial role in pathogen recognition activity [48]. The average number of motifs was greater in subfamilies VII, X, XI and XII, and a few genes in XIII, but there was no specific difference between RD and non-RD type kinases. Additionally, among the 303 *BrLRR-RLK* genes, only 234 genes contained a transmembrane domain, and regarding SPs, 276 genes contained one SP, whereas eight genes had two SPs, which were mostly found in subfamilies III and XI, and only one gene had three SPs (in subfamily III); the remaining eight genes lacked a SP (S2 Table).

### Classification of *B*. *rapa* LRR-RLRR- RLK genes according to their functions

Gene ontology annotations for the 303 *BrLRR-RLK* genes were retrieved using the Blast2Go software under the biological process, molecular function and cellular component categories. In the biological process category, the *BrLRR-RLK* genes were involved in 918 different processes with the highest number (249 genes) involved in cellular processes, followed by cellular metabolic processes (239), and phosphorylation and phosphate-containing compound metabolic processes (232) (S3 Table). Similarly, previous reports published on Arabidopsis suggest that LRR-RLK genes play roles in different cellular processes in relation to organ development and phosphorylation [10,49–51]. Furthermore, the *BrLRR-RLK* genes were associated with 74 different molecular functions, with the highest number of genes involved in transferase activity, transferring phosphorus-containing groups (268), kinase activity (267), binding (233), phosphotransferase activity, and ‘alcohol group as acceptor and protein kinase activity’ (241). Consistent with our observations, previous studies have reported the important roles of LRR-RLK genes in relation to metabolic activities [3,5,40,51]. In general, receptor kinases are important for cell-to-cell interactions, so many of these proteins are associated with the cell surface [5,37]. Here, the *BrLRR-RLK* genes were associated with 66 different cellular components including the membrane (251), ‘cell and cell part’ (221), and ‘cell periphery and plasma membrane’ (207) (S5 Table).

### Differential expression analysis of *B*. *rapa* LRR-RLK genes

**Fig 5:**
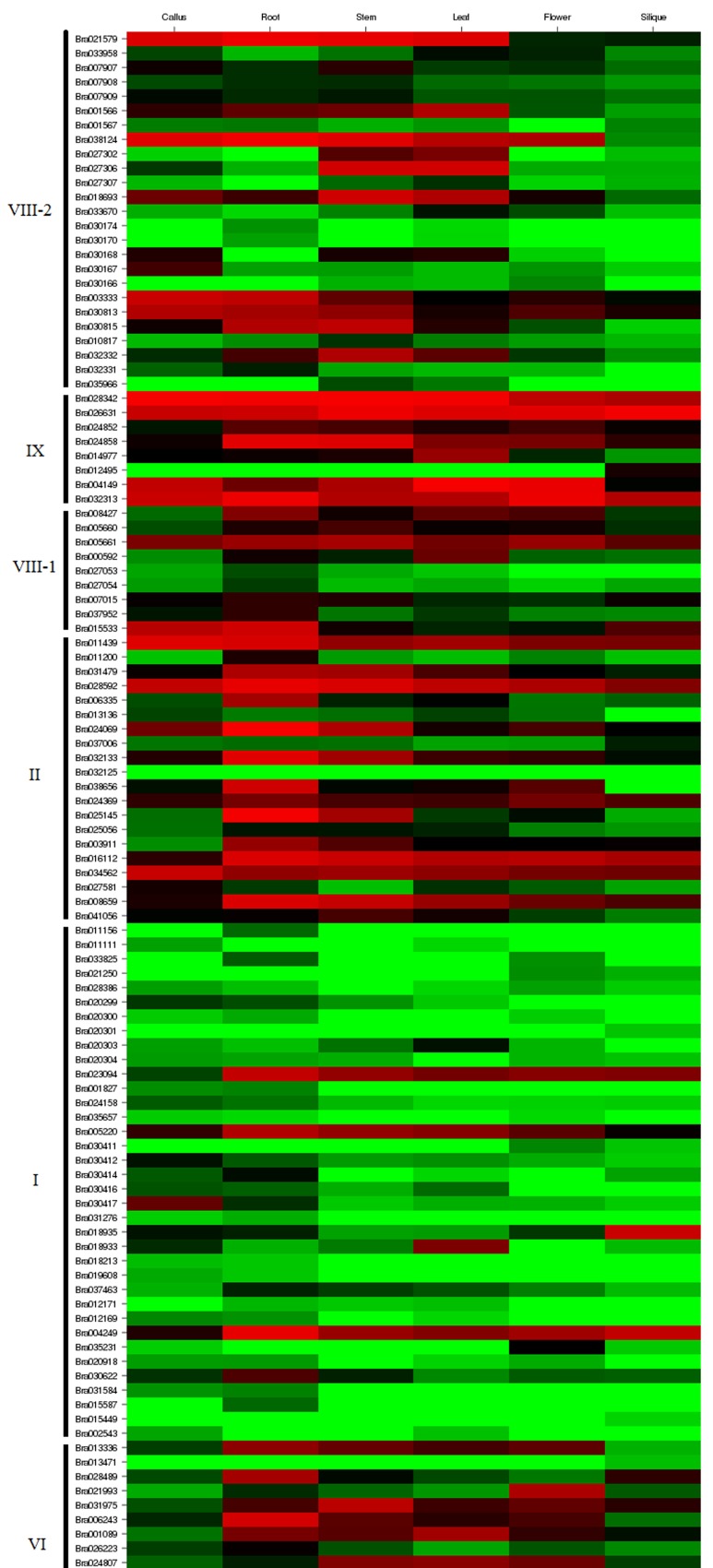
Transcript abundance of 303 *B*. *rapa* LRR-RLK genes. Microarray-based expression data for the genes downloaded from the Geo database were examined in six different tissues: callus, root, stem, leaf, flower and silique. The genes were grouped according to subfamily and the color scale at the bottom represents the expression values of the genes in the tissues. Please refer to S6 Fig for the full size version of Fig 5.

The expression abundance of a gene in different tissues can be used as a source of information for identifying its function in particular tissues; thus, expression data for the 303 *BrLRR-RLK* genes were extracted separately from a whole transcriptome dataset [21]. The genes were grouped according to subfamily, and a heat map was generated to analyze the expression variation among six different tissues of Chiifu—callus, root, stem, leaf, flower and silique (Fig 5). Furthermore, a few *BrLRR-RLK* genes were named after their *A*. *thaliana* homologs (S2 Table). Numerous expression variations were observed in the heat map of *BrLRR-RLK* genes so these genes were clustered into groups according to their expression levels. In addition to silique, the genes *BrLRR-RLK34*, *207* and *224* of subfamily VIII-2 had detectable differential expression in all the tissues whereas the remaining genes of this subfamily have failed to express in any of the tissues so the expression results can be depicted as may be they have functional roles during different developmental stages (Fig 5). The genes *BrLRR-RLK245*, *248* and *261* of subfamily VI and *BrLRR-RLK26*, *64*, *212* and *258* of subfamily XI had a high expression level in all tissues except that *BrLRR-RLK245* and *212* had moderate expression in the silique. Jones et al. [52] reported that *MRH1* is mainly involved in root hair elongation during tip growth in Arabidopsis; similarly in our results, a homolog of *MRH1*, *BrLRR-RLK12* of subfamily VI, had high expression in the root compared with other tissues. The genes of subfamily VIII-1 showed detectable to high expression abundance in all the tissues except for *BrLRR-RLK249*, which was not expressed in the flower or silique.

Generally, *SERK* genes function in anther development, male gametophyte maturation and disease resistance, whereas *NIK* genes function in signaling during virus infection [8,53,54]. Here, the genes *BrLRR-RLK95* (*SERK4*) and *168* of subfamily II were only expressed in siliques, *BrLRR-RLK110* was not expressed in any tissue and most of the remaining genes of this subfamily had detectable expression variation among all the tissues. In subfamily I, the genes *BrLRR-RLK84*, *126* and *215* had moderate to high expression in all tissues but the remaining genes of this subfamily had undetectable or no expression in a few tissues; moreover, the *MEE* and *RHS* genes of this subfamily are involved in embryo and root hair development [55,56] (Fig 5). A low to moderate expression level was observed for all three genes of subfamily XIV; conversely, in subfamily IV, four genes had moderate to high expression levels in most of the tissues. Among the 13 *BrLRR-RLK* genes of subfamily V, most showed detectable transcript abundance in all six different tissues; additionally, their homologous genes *SRF1–8* and *SUB/SRF9* of *A*. *thaliana* play major roles in leaf, stem, flower and inflorescence morphology and are also involved in cell wall biology, basic cell processes and abiotic stress [11,57]. Among the 52 genes of subfamily III, most had high expression levels in vegetative tissues like roots, stems and leaves compared with callus, flowers and siliques (Fig 5). Few homologs of the subfamily III genes have been published. Valon et al. [58] showed that *TMKL1* is transcriptionally active in different organs and also regulates the development of silique maturation. Similarly, *AtPRK2* is involved in pollen tube growth [59] and *IMK* functions in cell proliferation and cell fate determination [30].

In general, the subfamily XII genes *EFR* and *FLS2* are up-regulated during biotic stress [41]. Accordingly, all 25 genes of subfamily XII had detectable expression or a complete absence of transcripts in most of the tissues. In subfamilies XI, VII, XIII and X, most of the genes showed high expression levels in roots, stems and leaves, and in addition, a few genes also showed moderate to high expression levels in callus, flowers and siliques (Fig 5). Their homologous genes are involved in different functions: *CLV1*, *BAM1*, *BAM2* and *BAM3* in cell differentiation and control of size in the shoot and floral meristem, *GSO* in normal development of the epidermal surface of embryos, *HSL2* in flower abscission, *TOAD2* and *RPK1* in embryonic pattern formation, *FEI2* in synthesis of cell wall components, *ERL1* and *ERL2* in promoting organ growth and flower development and *BRL1*, *BRL3* and *BRI1* in normal vascular differentiation in Arabidopsis shoots [10,49,60–65]. Likewise, *SOBIR1*, *PEPR1*, *PEPR2* and *PSKR1* are mainly involved in defense responses, *BRI1* and *BAK1* in brassinosteroid signaling, and *BIR1* in plant resistance [14–17,66]. These expression data may be a valuable resource for further investigation of the functional significance of the *BrLRR-RLK* genes in *B*. *rapa*.

### Biochemical analysis of *BrLRR- RLK* genes

For biochemical analysis, we created 11 *BrLRR-RLK* constructs with the cytoplasmic domain, which consisted of a juxtamembrane domain, kinase domain and C-terminal region. Previous studies suggested that Arabidopsis *BAK1* identifies and phosphorylates *E*. *coli* proteins *in vitro* and a wide range of cytoplasmic domains including the juxtamembrane domain, and the C-terminal region regulates *BAK1* kinase activity and specificity through auto-phosphorylation and trans-phosphorylation [67]. We transformed Flag-tagged recombinant protein expression constructs for LRR-RLK cytoplasmic domains into BL21 *E*. *coli* cells and then performed comparative biochemical assays with the recombinant proteins. The phosphorylation levels of the expressed recombinant proteins were assessed with phosphorylation-specific antibodies, anti-phosphothreonine and anti-phosphotyrosine.

**Fig 6:**
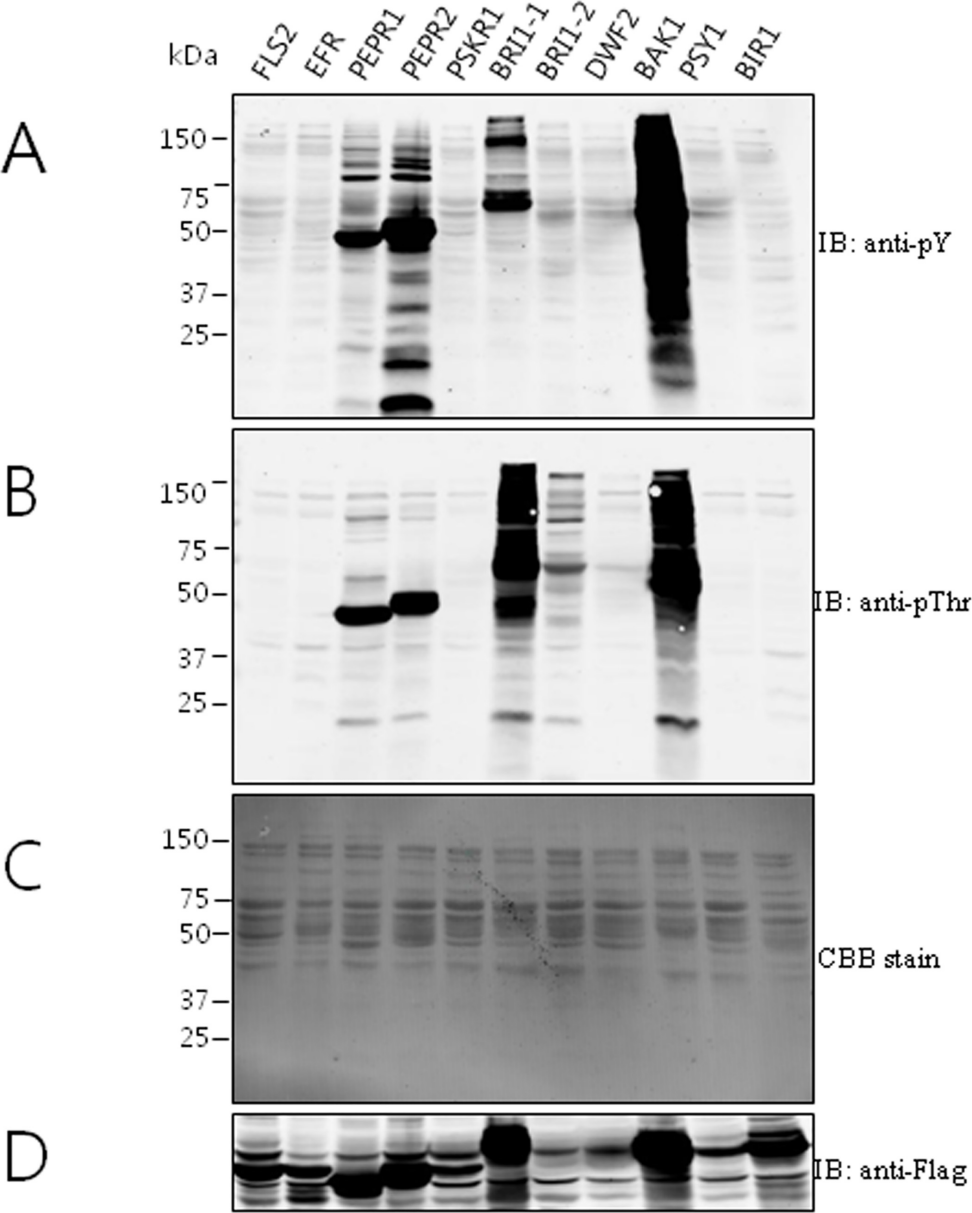
Auto-phosphorylation of recombinant *BrLRR- RLK’s* in *E*. *coli*. (A) Anti-phosphotyrosine immunoblots showing auto-phosphorylated proteins. (B) *BrLRR-RLK* genes showing auto-phosphorylation on anti-phosphothreonine residues. (C) Coomassie Brilliant Blue (CBB) staining of total extracted crude proteins. (D) Recombinant *BrLRR-RLK* protein expression levels were detected with an anti-FLAG antibody.

The expression levels of the 11 recombinant LRR-RLK proteins were detected with anti-Flag. As shown in Fig 6, seven LRR-RLKs showed auto- and trans-phosphorylation activity. Even though RD kinases have auto-phosphorylation ability, some of the RD kinases (*BrPSKR1* and *BrPSY1*) in our analysis did not phosphorylate tyrosine and/or threonine residues. This might have been because of a lack of catalytic activity at the target kinase [68]. Coomassie brilliant blue (CBB) staining of the total recombinant *E*. *coli* crude extract showed the protein expression. Interestingly, *BrBAK1* had strong auto-phosphorylation and trans-phosphorylation of tyrosine and threonine residues compared with *AtBAK1* in previous studies [69,70]. These data suggest that *BrBAK1* has much stronger kinase activity, which might be a very interesting result because the *BrBAK1* signaling pathway is involved in plant growth and development, plant innate immunity, programmed cell death, and other novel mechanisms with a different pattern compared with *AtBAK1*. Another interesting result was that the highly accumulated *BrBAK1*, *BrBRI1*, *BrPEPR1* and *BrPEPR2* proteins showed activity with both anti-phosphotyrosine and anti-phosphothreonine antibodies, indicating that they might have dual-specificity kinase activity.

**Fig 7:**
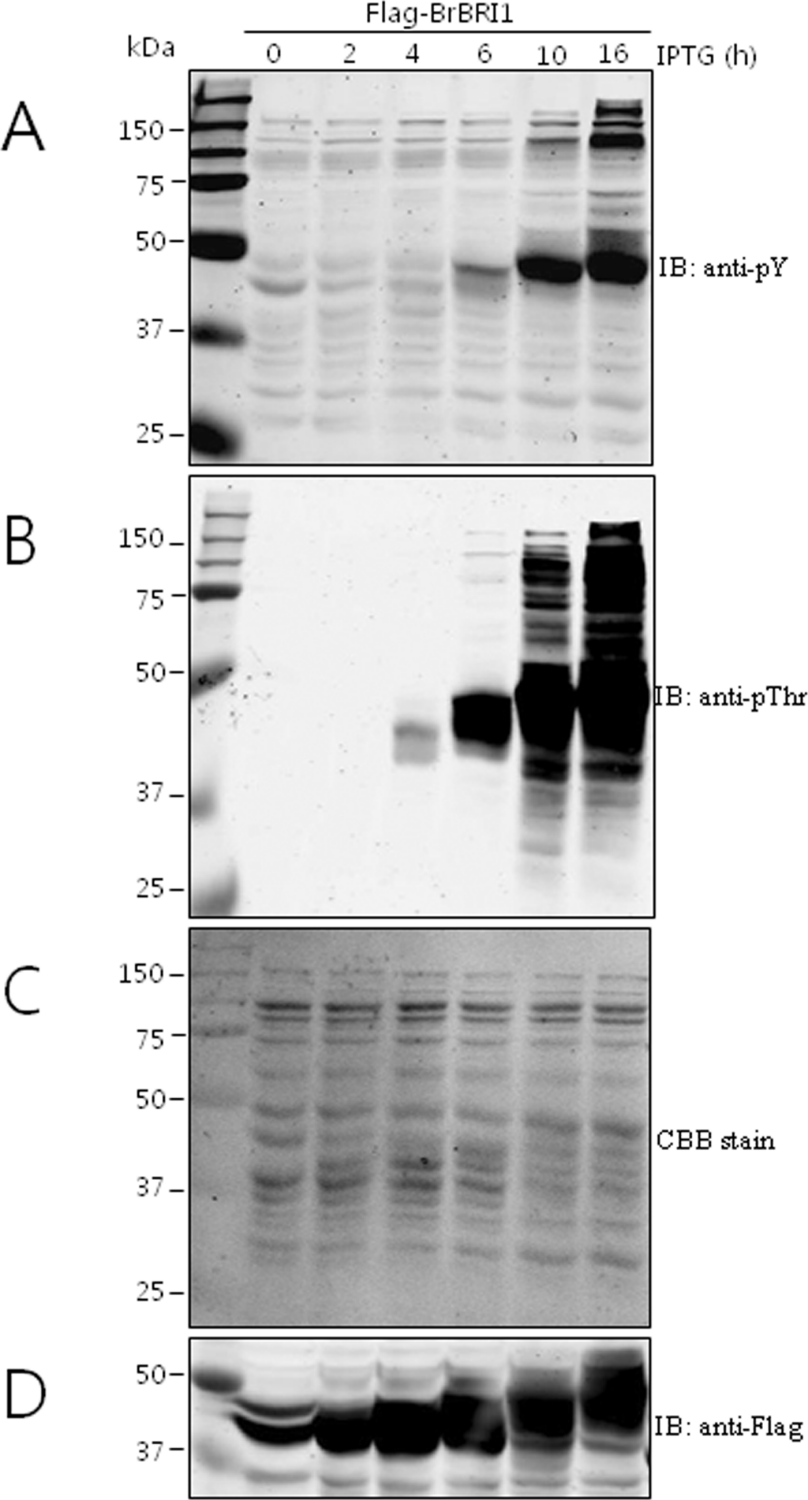
Immunoblot analysis of FLAG-*BrBRI1* kinase phosphoprotein production over a time course.

To further confirm the auto-phosphorylation of the Flag-*BrBRI1* receptor kinase, we induced the recombinant protein in *E*. *coli* cells and analyzed the protein over a time course of incubation after adding IPTG. As shown in Fig 7, Flag-*BrBRI1* auto-phosphorylation showed a gradual increase from 6 to 16 h (approx. size 45 kDa and 150 kDa) and more *E*. *coli* proteins were phosphorylated by the Flag-*BrBRI1* receptor kinase. Similarly, in anti-phosphothreonine analysis, auto-phosphorylation was observed from 4 h and showed the highest signal intensity at 16 h at around 50 kDa; in addition, several *E*. *coli* proteins were trans-phosphorylated. Compared with the phosphorylation of *BrBRI*-Flag with anti-phosphothreonine at 4 h, tyrosine auto-phosphorylation was observed at 6 h (Fig 7). These data suggest that specific threonine phosphosites were phosphorylated faster than specific tyrosine phosphosites on *BrBRI1 in vitro*. Similar to our results, previous research also proved that *AtBRI1* kinase has the ability to phosphorylate at serine/threonine and tyrosine residues, which helps in different functions related to plant growth and development [71,72]. This work provides a comprehensive analysis of the broad role of Tyr and Thr phosphorylation for the function of *B*. *rapa LRR-RLK*s.

## Conclusion

Our results for the *BrLRR-RLK* gene family suggest that duplications led to the expansion of the gene family. Comparative phylogenetic analysis allowed us to predict sequence similarities and identify the genes specific to each subfamily and the variations among monocot and dicot species (*A*. *thaliana*, *B*. *rapa*, *O*. *sativa* and *P*. *trichocarpa*). Motif and structural analysis showed motif conservation and intron/exon pattern variations among the subfamilies. In addition, gene ontology and expression analysis revealed various functional roles and tissue-specific expression variations. Finally, biochemical analysis demonstrated the type of receptor kinase activity occurring in cells. These results will be a major resource for studying the adaptive evolution, molecular mechanisms and functions of LRR-RLKs in the near future.

## Supporting Information

S1 Fig Rectangular phylogenetic tree of LRR-RLKs from *B*. *rapa*, *A*. *thaliana*, *O*. *sativa* and *P*. *trichocarpa*. The tree was generated using MEGA5.0 through the neighbor-joining method with 1000 bootstrap iterations and the genes were divided into 14 clusters (I to XIV, indicated in different colors).
(TIF)

S2 Fig Rectangular phylogenetic tree of LRR-RLKs from *B*. *rapa* and *A*. *thaliana*. The tree was generated using MEGA5.0 through the neighbor-joining method with 1000 bootstrap iterations.
(TIF)

S3 Fig *B*. *rapa* phylogenetic analysis of LRR-RLK genes. The tree was generated using MEGA5.0 through the neighbor-joining method with 1000 bootstrap iterations. The 14 clusters (I to XIV) are indicated in different colors.
(TIF)

S4 Fig Amino acid conservation of the 25 motifs distributed in the *B*. *rapa* LRR-RLK genes. The motifs were identified using the MEME search tool.
(TIF)

S5 Fig This is the full size version of Fig 4. (TIF)

S6 Fig This is the full size version of Fig 5
(TIF)

S1 Table Gene-specific primers used in this study. (DOC)

S2 Table Detailed information and characteristics of the identified *BrLRR-RLK* genes. (A) RD and (B) non-RD type.
(XLSX)

S3 Table Details of the LRR-RLK gene duplications identified in the genome of *B*. *rapa*. (A) Paralogical copies of *B*. *rapa LRR-RLK* genes and (B) Tandem and Segmental duplications of *B*. *rapa LRR-RLK* genes.
(XLSX)

S4 Table Amino acid sequence alignment for RD and non-RD type LRR-RLK genes identified in *B*. *rapa*. (DOCX)

S5 Table Gene Ontology of the 303 *BrLRR-RLK* genes. (A) Biological process, (B) molecular function and (C) cellular localization.
(XLSX)
