# Supplementary figures and images for: Genomic and Post-Translational Modification Analysis of Leucine-Rich-Repeat Receptor-Like Kinases in Brassica rapa
Source: PLoS One. 2015 Nov 20;10(11):e0142255. doi: 10.1371/journal.pone.0142255 (PMC4654520; doi:10.1371/journal.pone.0142255)

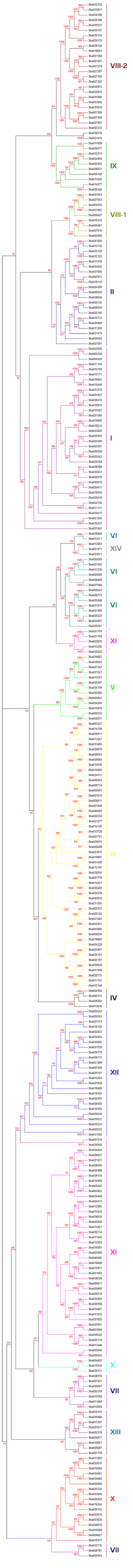

Supplement: S3 Fig — The tree was generated using MEGA5.0 through the neighbor-joining method with 1000 bootstrap iterations. The 14 clusters (I to XIV) are indicated in different colors. (TIF) [file pone.0142255.s003.tif]

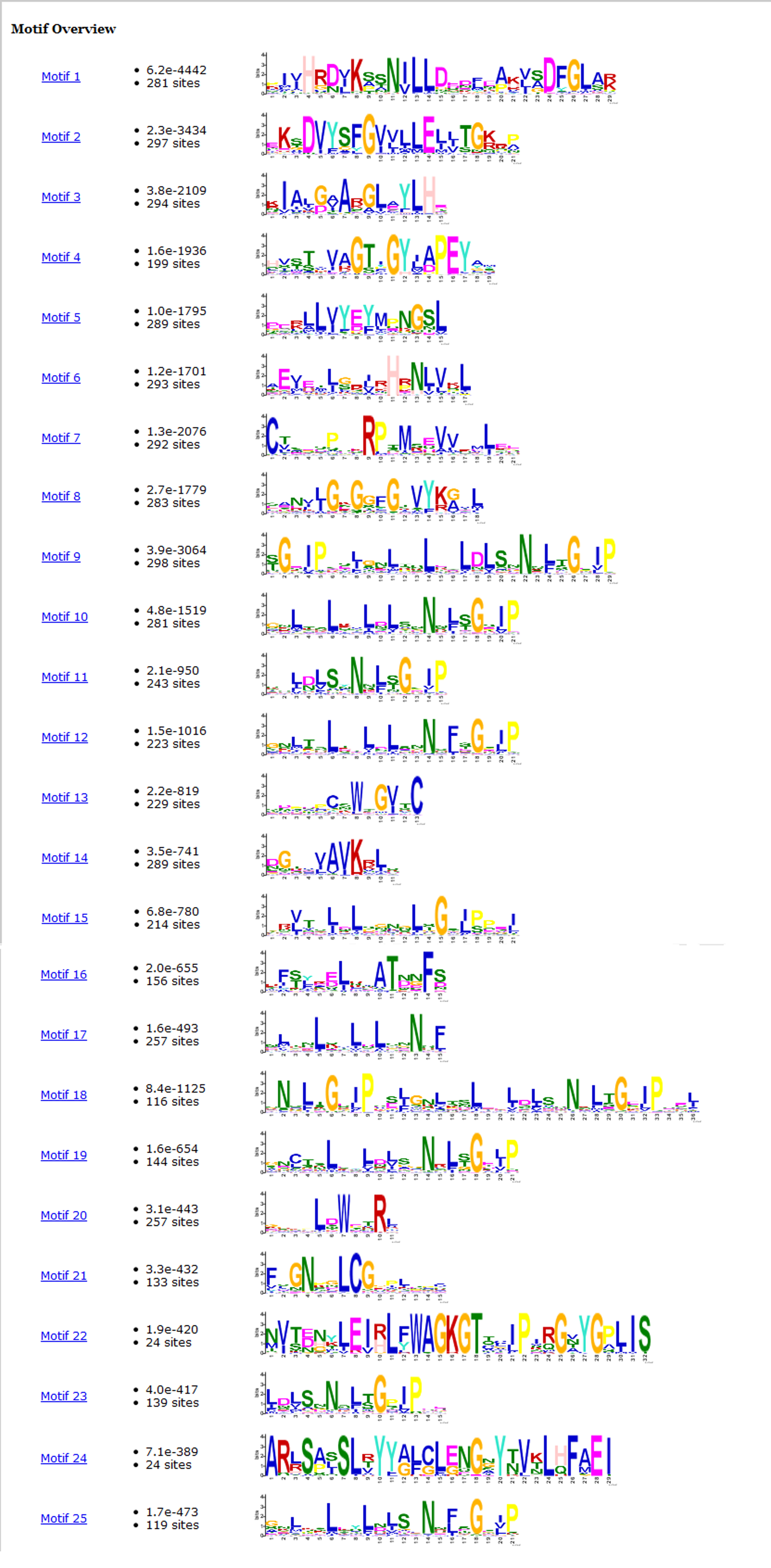

Supplement: S4 Fig — The motifs were identified using the MEME search tool. (TIF) [file pone.0142255.s004.tif]

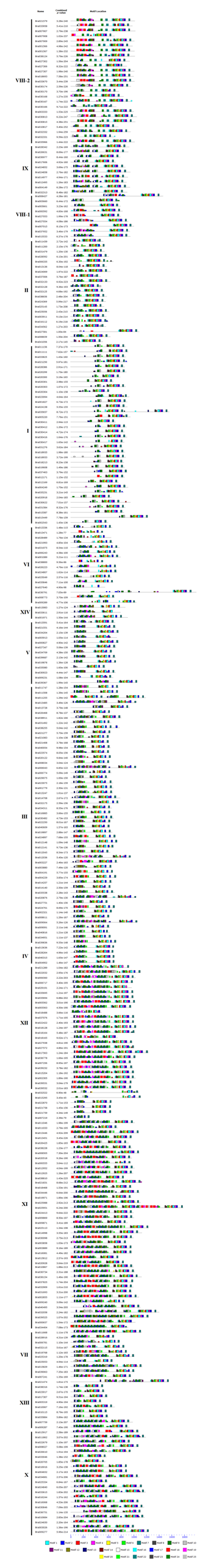

Supplement: S5 Fig — (TIF) [file pone.0142255.s005.tif]

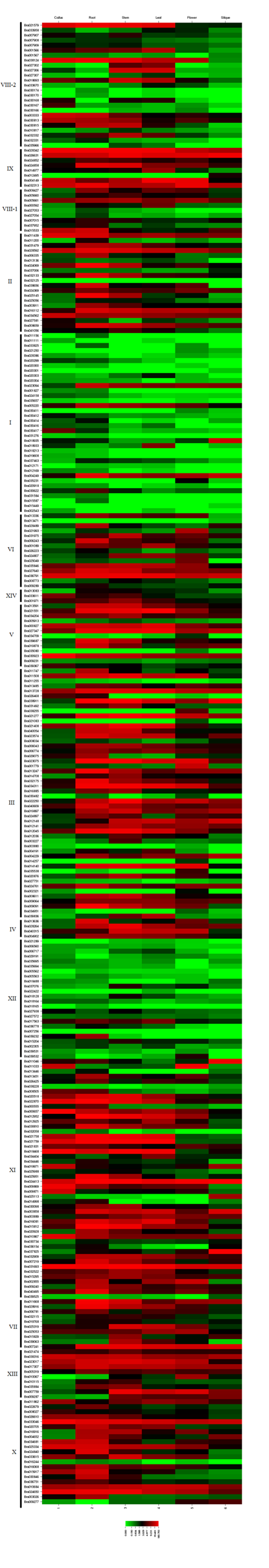

Supplement: S6 Fig — (TIF) [file pone.0142255.s006.tif]
