# Supplementary material for: Genomic and Post-Translational Modification Analysis of Leucine-Rich-Repeat Receptor-Like Kinases in Brassica rapa
Source: PLoS One. 2015 Nov 20;10(11):e0142255. doi: 10.1371/journal.pone.0142255 (PMC4654520; doi:10.1371/journal.pone.0142255)
Supplement: S1 Table — (DOC) [file pone.0142255.s007.doc]

S1 Table. Gene-specific primers used in this study.

| **Gene name** | **Primer Sequences** | | **Applications** |
| --- | --- | --- | --- |
|  | **Forward** | **Reverse[5’-3’]** |  |
| Bra017563 | ATGACTTCACTCTCAAAGACAACTTTG  CTGACTCGAGCTCACCTGCTGCAGAAAAAAG | TTACACTTCTAAATCACCAATACGATCAGG  GACCCGGGTTACACTTCTAAATCACCAATACGATC | Full length PCR(pENTRY-TOPO vector cloning)  Recombinant Flag(pFLAG-MAC vector cloning) |
| Bra002305 | ATGAAGCCGTTTCTTTCAATTGCTC  GATCGAATTCAAGAAGAAGAGGGACAAGAGGA | CTACATTGTATGCATGTCCGCGC  GAGGTACCCTACATTGTATGCATGTCCGC | Full length PCR(pENTRY-TOPO vector cloning)  Recombinant Flag(pFLAG-MAC vector cloning) |
| Bra003858 | ATGCTGGAGAATCTCACTGACTTG  CTGACTCGAGATCTGCCTACGCCGTCGT | TTAGCGAACTGAACCAGAGGAG  GATACCCGGGTTAGCGAACTGAACCAGAGGA | Full length PCR(pENTRY-TOPO vector cloning)  Recombinant Flag(pFLAG-MAC vector cloning) |
| Bra025951 | ATGATGAATATTGGGTTAGTTGGAATTAC  CTGACTCGAGACCTGCTGCAAAAGAGGAG | CTATTGAACTGAACCAGAAGTGCTTCTTACG  ACCCGGGCTATTGAACTGAACCAGAAGTGC | Full length PCR(pENTRY-TOPO vector cloning)  Recombinant Flag(pFLAG-MAC vector cloning) |
| Bra026610 | ATGCGTGTTCATCGCTTGTG  GATCGAATTCCGTGCGCGTAGACGGTCAG | CTAGACATGATCAAGCCAAGAGACC  GACCCGGGCTAGACATGATCAAGCCAAGAG | Full length PCR(pENTRY-TOPO vector cloning)  Recombinant Flag(pFLAG-MAC vector cloning) |
| Bra011862 | ATGAACACTTTTCTAAGCGTCTTTC  CTGACTCGAGGGTAGAGAGATGAGGAAGAGA | TCAGAATTTTCCTTCGGGAACTTC  GACCCGGGTCAGAATTTTCCTTCGGGAACTTC | Full length PCR(pENTRY-TOPO vector cloning)  Recombinant Flag(pFLAG-MAC vector cloning) |
| Bra033615 | ATGAAGACTTTTCCAAGCTTCTTTC  GATCGAATTCGGTAGAGAGATGAGGAAGAGA | TCAGAATTTTCCTTCGGGAACTTC  GACCCGGGTCAGAATTTTCCTTCGGGAACTTC | Full length PCR(pENTRY-TOPO vector cloning)  Recombinant Flag(pFLAG-MAC vector cloning) |
| **Gene name** | **Primer Sequences** | | **Applications** |
|  | **Forward** | **Reverse [5’-3’]** |  |
| Bra010684 | ATGAGAATGAAAACTTTCCCAACCTTC  GATCGAATTCGGTAGAGAGATGAGGAGGC | TCAAAACTTTCCTTCAGGAACTTCTTTAATAC  GACCCGGGCAAAACTTTCCTTCAGGAACTTCTTTAATAC | Full length PCR(pENTRY-TOPO vector cloning)  Recombinant Flag(pFLAG-MAC vector cloning) |
| Bra011439 | ATGGAACGAAGATTGATGAAGATAGC  CTGACTCGAGGCTCTGTGGCGAAGGAAAA | TTATCTTGGACCCGAGGGGTAATC  GACCCGGGTTATCTTGGACCCGAGGGG | Full length PCR(pENTRY-TOPO vector cloning)  Recombinant Flag(pFLAG-MAC vector cloning) |
| Bra016068 | ATGATTGACGAGAAGAAGATGAGATC  GATCGAATTCTCAAAGAGGAGAGTAAACCCAG | TTACTTTGTCTCTTCTTCTTCTTCAGCTTC  GACCCGGGTTACTTTGTCTCTTCTTCTTCTTCAGC | Full length PCR(pENTRY-TOPO vector cloning)  Recombinant Flag(pFLAG-MAC vector cloning) |
| Bra020705 | ATGATGGTGAGTAGTAGAGTTTTGAGC  CTGACTCGAGTACTTCCGTAGATTGGCTGTTG | TCAGCGAACTATCATCTCTTCAATG  GACCCGGGTCAGCGAACTATCATCTCTTCA | Full length PCR(pENTRY-TOPO vector cloning)  Recombinant Flag(pFLAG-MAC vector cloning) |
