# Supplementary material for: Genomic and Post-Translational Modification Analysis of Leucine-Rich-Repeat Receptor-Like Kinases in Brassica rapa
Source: PLoS One. 2015 Nov 20;10(11):e0142255. doi: 10.1371/journal.pone.0142255 (PMC4654520; doi:10.1371/journal.pone.0142255)
Supplement: S4 Table — (DOCX) [file pone.0142255.s010.docx]

| **Gene** | **BRAD ID** | **At.ID** | **Sub-Domain** |
| --- | --- | --- | --- |
| *FLS2* | Bra022032 | AT5G46330.1 | ＰＩＶＨ**ＣＤ**ＬＫＰＡ |
| *EFR* | Bra006560 | AT5G20480.1 | ＰＶＡＨ**ＣＤ**ＬＫＰＳ |
| *EFR* | Bra002305 | AT5G20480.1 | ＱＩＡＨ**ＣＤ**ＬＫＰＳ |
| *BIR1* | Bra020705 | AT5G48380.1 | ＲＩＩＨ**ＲＮ**ＩＳＳＫ |
| *PEPR1* | Bra003858 | AT1G73080.1 | ＰＩＶＨ**ＲＤ**ＩＫＰＥ |
| *PEPR2* | Bra025951 | AT1G17750.1 | ＰＩＩＨ**ＲＤ**ＩＫＰＥ |
| *PSKR1* | Bra026610 | AT2G02220.1 | ＨＩＬＨ**ＲＤ**ＩＫＳＳ |
| *BRI1* | Bra010684 | AT4G39400.1 | ＨＩＩＨ**ＲＤ**ＭＫＳＳ |
| *BAK1* | Bra034562 | AT4G33430.2 | ＫＩＩＨ**ＲＤ**ＶＫＡＡ |
| *PSY1* | Bra008027 | AT1G72300.1 | ＨＩＶＨ**ＲＤ**ＩＫＳＳ |

S4 Table. Amino acid sequence alignment for RD and non-RD type LRR-RLK genes identified in *B. rapa.*
